# A Checklist for Involving Patients in Educational Activities

**DOI:** 10.1111/tct.70282

**Published:** 2025-11-16

**Authors:** Mandy Young, Cathy Kline, Angela Towle

**Affiliations:** ^1^ Patient & Community Partnership for Education, Office of UBC Health University of British Columbia Vancouver Canada

## Abstract

Teachers in the health professions increasingly see the benefits of involving patients in their educational activities and are looking for good practice guidelines on how best to do this, especially when they lack experience. Patient partners say that they often do not get the information they need in order to understand expectations and prepare effectively for their teaching role. In collaboration with patient partners, we developed a checklist in the form of a parallel document, one side for instructors and one side for patient partners. The checklist is in four parts and covers the things for teachers and patients to do before, during and after an educational activity. The checklist has been used by patient partners and instructors in a wide range of health professions at two institutions. It provides a concise and comprehensive reminder for instructors, empowers patients to ask for the information they need and is a template that can be customised for different contexts.

## 1 Introduction

The benefits of involving patients in the education of health professional students have been well documented [1, 2, 3, 4]. Patients have many kinds of unique experiential knowledge gained through lived experience that can complement the professional knowledge of educators [5]. However, patients need to be able to share these experiences with students in ways that promote learning, and teachers require guidance on how to work in partnership with patients and support them in their role, especially if they have not previously worked with patients in a classroom setting [6, 7].

*Teachers require guidance on how to work in partnership with patients and support them in their role*.

In a focus group study we conducted in 2002, patients with extensive experience in health professional education (HPE) identified the need to prepare for participation on the part of both patients and teachers [8]. Preparation included clear communication of expectations, information and orientation sessions and knowledge of the curriculum. Participants also identified that sharing personal experiences makes patients vulnerable and that instructors leading or facilitating educational sessions need to be prepared for their role in creating a relationship and a welcoming environment [8]. These findings have been borne out in other studies [7, 9, 10].

Most practical guidance on patient involvement is designed to help healthcare leaders and managers, practitioners, patients and families collaborate more effectively in health service delivery, quality improvement and research. In the context of HPE, practical guidance has focused more at the institutional or programme level [7, 11, 12]. There is limited practical guidance for individual teachers who want to involve patients in an educational activity, perhaps for the first time. In this article, we focus on the involvement of patients who have extensive lived experience in the healthcare system, who are actively involved in healthcare improvement, who are aware they have this position (referred to as patient partners) and who share their lived experiences with students in the classroom rather than the clinic setting.

As patient involvement in HPE increased at our institution, we noted specific challenges that patient partners and faculty face in effectively preparing for, and participating in, educational sessions. We heard from students and patients that sometimes neither party knew how the patients' story connected with what the students were learning. Patient partners wanted to be prepared so they could shape their experiences to meet student learning objectives and be good educators. We identified two problems that could be easily addressed: (i) a lack of clarity regarding expectations and session objectives for patient partners and (ii) variability in the information provided to patient partners so that they can prepare for sessions. This led us to create a checklist for preparation for use by patients and instructors. The purpose of the checklist is to ensure that patients have what they need to participate confidently, responsibly, professionally and effectively in HPE activities. The checklist is in the form of a parallel document for instructors and patients to ensure all parties are well prepared and can collaborate seamlessly to enhance the learners' experience.

*We noted specific challenges that patient partners and faculty face in effectively preparing for, and participating in, educational sessions*.

## 2 Steps in Developing a Checklist

In May 2024, eight patient partners, including author MY, participated in a workshop to brainstorm ways to support new patient partners joining our network. We divided participants into two groups, one of experienced and one of relatively new, patient partners, to identify what supports are needed in order to orient patients to their roles. Drawing from their own experiences, participants shared what had worked well, what had not and what they wished they had known when starting out. The ideas generated during the workshop were organised and synthesised into key themes which formed the basis of an initial draft. We circulated the draft document to workshop participants and followed up with a virtual meeting to gather their feedback. During this meeting, participants recommended developing a checklist in the form of a parallel document, one side for instructors and one side for patient partners. Based on the feedback, we revised the checklist and recirculated it for final review and approval. We then shared the document with community partner organisations, patient partners in the health authorities and faculty members at our institution. Additional refinements were made based on their input. Ethics approval was not required by our institution as this was a quality improvement project. The quotes we use in this paper are not attributable to any individual and were provided in the context of feedback to improve the checklist.

*Participants recommended developing a checklist in the form of a parallel document, one side for instructors and one side for patient partners*.

The final checklist is in four parts:

- Before the event: orientation and preparation checklist (Table 1)
- Final details checklist (Table 2)
- Checklist for the day of the event (Table 3)
- Checklist for after the event (Table 4)

**TABLE 1:** Before the educational activity: Orientation and preparation checklist.

	For instructor	For patient partner
Learning objectives	Provide a description/lesson plan of the planned educational activity along with its learning objectives.	Make sure you understand the objectives or ask for clarification.
	State what year students are and what programme they are studying so the patient partner understands the audience.	Share your story/perspective in a way that it is most applicable to the students/programme.
Role expectations and requirements	Provide the patient partner with an outline of the lesson plan. Specify the topics and how long the patient partner is expected to speak.	Prepare your key perspectives/story for the experience using the prompts in the lesson plan based on the time permitted.
Logistics	Identify technology and teaching materials needed. Will the patient partner need to supply photos or short paragraph/bio to enhance a slide show? Offer assistance with this.	Are you tech savvy? Be sure to ask for whatever you require: microphones for in‐person activities or technology support with virtual activities.
	Decide in advance who will keep time and how to signal when to wrap up.	Is it easier for you to set your own timer?
	How long will the Q&A session last, who will facilitate and how will questions the patient partner prefers not to answer be handled?	Refer to the storytelling documents to craft your story—Institute for Patient and Family‐Centered Care provides examples.
	Set out expectations for the patient partner regarding preparatory meetings, rehearsals or review of materials.	Set aside time to prepare based on overview of the activity and attend the preparatory meeting.

*Note:* One week before the activity: (1) Confirm the patient partner is still available for the scheduled engagement. (2) Send final meeting details: (a) For virtual activities, send the online meeting link. (b) For in‐person activities, confirm detailed location directions with a visual map attached highlighting the presentation room number, building name, bus routes, parking locations, entrances, including the specified accessible entrance information, and who will meet the patient partner and where.

**TABLE 2:** Final details checklist.

In‐person or virtual activities	For instructor	For patient partner
Confirmations	Send invitation once date/time is confirmed.	Confirm you have the date and time in your calendar.
Location details (for in‐person activities)	Provide a campus map, building name and room number.	Familiarise yourself with the location ahead of time.
Access	Provide public transit information or parking location and information about parking (e.g., parking passes/reimbursement).	Decide if you will take public transit or drive. Confirm parking arrangements.
	Identify accessible entrances.	Where will you enter the building from?
Contact person details	Designate a primary contact person, such as the course lead, to answer questions and resolve problems.	Do you know who the primary contact person is?
	Provide information for general and technical support.	Do you feel all your questions have been answered?
	Consider exchanging contact phone numbers.	Do you feel comfortable sharing your phone number?
Briefings/debriefings	Is there a preactivity rehearsal to review the session flow?	Would it be helpful to run through the activity ahead of time?
	Connect with the patient partner at the end of the activity to ensure their safety and comfort after telling their story.	You have just retold your personal story, and you may have some emotions that are surfacing for you. Use this time to debrief.
Size and composition	Will the activity be in small breakout groups or a lecture style?	Do you feel comfortable with the format of the activity?
	Provide the size of the audience, a description of the room, and determine whether microphones are needed.	Do you feel comfortable with the group size and format of the activity?
Compensation	Ensure the patient partner is set up with financial services to receive payment.	Have you filled out the necessary paperwork to receive payment?
	Confirm the amount the patient partner will receive honoraria.	Are there any personal financial implications you should be mindful of when accepting compensation?

**TABLE 3:** Checklist for the day of the activity.

	For instructor	For patient partner
In‐person activity		
Welcome the patient partner	Offer a warm welcome and provide water.	Take time to relax—You are about to tell your personal story
	Identify the location of the washrooms and nearest exit.	Are there any other spaces you would like to know the location of?
	Place a box of tissues close to where they will be speaking.	It is always OK to show emotion—Your story is personal.
Logistics	Ask about any accommodations they need to present confidently.	Do you have everything you need?
	Review any technical aspects (e.g., microphone, presentation slides and lectern controls) to ensure their ease and comfort.	Are you comfortable with the technical aspects of the session?
	Provide information for general and technical support.	Do you feel all your questions have been answered?
Virtual activity		
Welcome the patient partner/logistics	Ask about any accommodations they need to present confidently.	You will be telling your personal story and you may have some emotions that are surfacing—Use this time to relax.
	Review any technical aspects (e.g., video, audio settings, slide sharing, polls and virtual rooms) to ensure their ease and comfort.	Are you comfortable with the technical aspects of the activity?
	Review who introduces participants, orients the audience, monitors questions and moderates Q&As.	Are you confident about the roles you will be taking?

**TABLE 4:** Checklist for after the activity.

	For instructor	For patient partner
One day after	Send a thank you e‐mail or card to express appreciation for participation. Send a feedback survey. Set up a virtual debriefing to review the activity and share feedback.	Complete the feedback survey. Accept or decline the virtual debriefing meeting.
One week after	Share results of feedback survey with the patient partner. Provide any additional individual insight from your perspective for improvements.	Attend debriefing. Provide any additional individual feedback from your perspective for improvements.
	Confirm reimbursement is on the way and provide details.	Confirm once you have received reimbursement.
	Follow up to support the patient partner's safety and well‐being, ensuring they feel valued and heard.	Reach out to the instructor or community engagement liaison for additional follow‐up support.

## 3 Use of the Checklist: Feedback and Lessons Learned

In August 2024, we distributed the checklist to 39 instructors, programme administrators and patient partners. Some immediate responses were received by e‐mail. For example:

These are fantastic guides, such valuable resources. So many memories come back where I would have given my right arm if I'd known this or if my host would have known these things. (Patient Partner)

Thank‐You so much for sharing these documents with us—I've just reviewed them and found them excellent and really useful! (Director of Medical Curriculum)

Approximately 6 months after we began to use the checklist, we sent an e‐mail to all the instructors who had received the checklist to ask whether they had used it and, if so, what they had liked about it and their suggestions for improvement. E‐mail responses were received from 13 instructors and programme administrators. Seven said they had not had an opportunity to use it, either because they were still to plan their patient partner educational activity or had planned it before receiving the checklist. All, even those who had not used it, found the checklist useful, especially those who were engaging patient partners for the first time. Those who had more experience found it a useful reminder. We did not receive any neutral or negative comments. Some suggestions for improvement were made to customise the checklist for their particular contexts. The following quotes illustrate the responses we received from those who had used the checklist. We consider the checklist to be a living document and will continue to modify it based on feedback.

I liked that this single document provided a comprehensive summary of expectations for both the speakers and myself, and also provided some structure for our pre‐class planning meeting. One consideration is that I wasn't always sure I had modified/tailored the document in a way that was clearest to our patient partners … formatting the document to really highlight specifics for that event/class plan could be helpful. (Instructor, Genetics Counselling)

I found it a very valuable resource to structure my thinking in how best to support the patient partners coming in … Having the learning objectives and the possible discussion questions ahead of time allowed the patient partners to come in thinking about what some questions might be asked of them … As an addendum to the checklist, I would suggest discussing with all partners if they want to have photos present at their talk and if so, to arrange that ahead of time. (Instructor, Social Work)

I have used this checklist to onboard a patient partner for the [interprofessional education] session for Year 4 students in the MD Undergraduate program. The document is clear, detailed, and organized from beginning to end. Helpful outline of what items should be communicated first to the patient partners, and how to continue supporting throughout the process. The timeline checklist is also very much appreciated, particularly the details regarding resending the meeting link/location directions on the day of the event for easy reference to the Patient Partners. (Programme Administrator, Medicine)

Following the initial dissemination of the checklist, we have continued to find ways to put it in front of people more often. Every time we create a new version, we send it out to everyone as a reminder. We have embedded it into the e‐mail communication that connects patient partners we have recruited with the faculty lead for the session. We have not formally evaluated its use by patient partners, but anecdotally, we know that before we introduced the checklist, we received more queries from patients about the session. Now, patient partners use the checklist to get the information they need for themselves, for example, by asking the teaching team to set up a 15‐min meeting before the session.

*Patient partners use the checklist to get the information they need for themselves*.

We have learned the following lessons from our experiences so far.

- People who use the checklist pick and choose what they find to be most useful.
- Faculty prefer to have a checklist that is ‘theirs’; patients and programme administrators like having both the patient and faculty checklists in parallel.
- Having a parallel checklist reduces the power imbalance between patient partners and instructors as everyone has the same information.
- The use of the checklist by faculty requires a change of behaviour on their part and they need constant reminders for it to become part of their planning routine.
- The checklist empowers patient partners to get the information they need (e.g., learning objectives) to prepare for an educational activity.

*Having a parallel checklist reduces the power imbalance between patient partners and instructors as everyone has the same information*.

## 4 Summary of Key Ideas

The checklist was developed by patients for patients and instructors. While we continue to use the checklist in our own work, it has also been adapted by colleagues at another institution that engages patient partners in educating allied health professions not represented at our institution. We offer the checklist as a flexible organising tool that can be tailored to different contexts even as it continues to evolve. For example, some instructors have suggested format changes and requested that the checklist be available online. It can also serve as a template that instructors can customise to fit their specific institutional needs, such as adding information about locations or compensation or removing items that may not apply. Providing the checklist to patient partners empowers them to get the information they need to prepare effectively for an educational session.

*We offer the checklist as a flexible organising tool that can be tailored to different contexts even as it continues to evolve*.

## Author Contributions

**Mandy Young:** conceptualization, methodology, visualization, writing – review and editing. **Cathy Kline:** conceptualization, methodology, supervision, writing – review and editing. **Angela Towle:** conceptualization, methodology, writing – original and review and editing.

## Ethics Statement

Quality assurance and quality improvement projects are exempt from review by University of British Columbia Office of Research Ethics.

## Conflicts of Interest

The authors declare no conflicts of interest.

## Data Availability

The data that support the findings of this study are available from the corresponding author upon reasonable request.

## References

[1] A. Towle , C. Farrell , M. E. Gaines , et al., “The Patient's Voice in Health and Social Care Professional Education. The Vancouver Statement,” International Journal of Health Governance 21, no. 1 (2016): 1–8.

[2] S. W. Dijk , E. J. Duijzer , and M. Wienold , “Role of Active Patient Involvement in Undergraduate Medical Education: A Systematic Review,” BMJ Open 10, no. 7 (2020): e037217. 10.1136/bmjopen-2020-037217 PMC7389514 32718925

[3] M. Gordon , S. Gupta , D. Thornton , M. Reid , E. Mallen , and A. Melling , “Patient/Service User Involvement in Medical Education: A Best Evidence Medical Education (BEME) Systematic Review: BEME Guide No. 58,” Medical Teacher 42, no. 1 (2020): 4–16. 31518544 10.1080/0142159X.2019.1652731

[4] L. Nowell , B. Keogh , E. Laios , L. Mckendrick‐Calder , W. Molitor , and K. Wilbur , “Public Participation in Healthcare Students' Education: An Umbrella Review,” Health Expectations 27, no. 1 (2024): e13974. 39102698 10.1111/hex.13974 PMC10801288

[5] V. Dumez and A. L'Esperance , “Beyond Experiential Knowledge: A Classification of Patient Knowledge,” Social Theory and Health 22 (2024): 173–186.

[6] M. Cullen , C. Cadogan , S. George , et al., “Key Stakeholders' Views, Experiences and Expectations of Patient and Public Involvement in Healthcare Professions' Education: A Qualitative Study,” BMC Medical Education 22 (2022): 305. 35449105 10.1186/s12909-022-03373-z PMC9026974

[7] C. Eijkelboom , M. Brouwers , J. Frenkel , et al., “Twelve Tips for Patient Involvement in Health Professions Education,” Patient Education and Counseling 106 (2023): 92–97. 36266155 10.1016/j.pec.2022.09.016

[8] A. Towle , K. Ong , L. Wang , and C. C. Kline , “Patient/Public Perceptions on Engagement With a Medical School: What Needs to Happen to Support Authentic and Sustained Participation?,” Medical Teacher 46, no. 7 (2024): 963–970. 38071663 10.1080/0142159X.2023.2289843

[9] S. Alberti , P. Ferri , L. Ghirotto , et al., “The Patient Involvement in Nursing Education: A Mixed Methods Systematic Review,” Nurse Education Today 128 (2023): 105875. 37336122 10.1016/j.nedt.2023.105875

[10] K. Metersky , R. Rahman , and J. Boyle , “Patient‐Partners as Educators: Vulnerability Related to Sharing of Lived Experience,” Journal of Patient Experience 10 (2023): 1–4. 10.1177/23743735231183677 PMC10286174 37362248

[11] A. Towle and W. Godolphin , “Patients as Teachers: Promoting Their Authentic and Autonomous Voices,” Clinical Teacher 12 (2015): 149–154. 26009947 10.1111/tct.12400

[12] M. Brahmaputra , J. Scobie , K. Doyle , et al., “Twelve Tips for Engaging Students and Community Partners in Medical Education,” Medical Teacher 44 (2022): 1340–1346. 34634989 10.1080/0142159X.2021.1986625

